# Outbreak of acute larval cyathostominosis – A “perfect storm” of inflammation and dysbiosis

**DOI:** 10.1111/evj.13350

**Published:** 2020-10-06

**Authors:** Nicola Walshe, Grace Mulcahy, Fiona Crispie, Raul Cabrera‐Rubio, Paul Cotter, Hanne Jahns, Vivienne Duggan

**Affiliations:** ^1^ School of Veterinary Medicine University College Dublin Dublin Ireland; ^2^ Conway Institute of Biomedical and Biomolecular Research University College Dublin Dublin Ireland; ^3^ Teagasc Food Research Centre APC Microbiome Moorepark Ireland; ^4^ APC Microbiome Ireland Moorepark Ireland; ^5^ Vistamilk Moorepark Ireland

**Keywords:** horse, helminths, microbiota, cyathostomin, dysbiosis, outbreak, strongyles

## Abstract

**Background:**

Cyathostomins are prevalent and pathogenic intestinal helminths of horses, causing acute and chronic disease, including acute larval cyathostominosis, which has a mortality rate of 50%. Factors determining individual susceptibility to acute larval cyathostominosis are unknown. Investigation of these factors could lead to novel treatment and prevention strategies.

**Objectives:**

To investigate clinicopathological and faecal microbiota changes associated with disease in individual horses in an acute larval cyathostominosis outbreak.

**Study design:**

Case series.

**Methods:**

The study population was a herd of 23 mixed breed horses in Ireland. The outbreak occurred in November 2018. Fourteen horses were clinically affected. Clinical status was monitored and recorded. Blood and faecal sampling allowed clinicopathological, faecal 16s rRNA gene sequencing and faecal egg count analyses.

**Results:**

Two horses were euthanised, whilst 12 recovered. Common clinical signs included loose faecal consistency, weight loss and pyrexia. Consistent clinicopathological findings were borderline anaemia, leucocytosis, thrombocytosis, hyperfibrinogenaemia, hyperglobulinaemia and a reverse A: G ratio. Decreased alpha‐diversity of the faecal microbiota and greater relative abundance of the genus *Streptococcus,* class Bacilli, order Lactobacillales and family Streptococcaceae, and family Prevotelleceae was found in clinically affected horses compared to their clinically normal cohorts. An increase in obligate fibrolytic bacteria was seen in the clinically normal group compared to the clinical group. Histopathological findings of the colon and caecum revealed a severe necrotising typhlocolitis associated with cyathostomin larvae and bacterial overgrowth in the mucosa of the large intestine.

**Main limitations:**

The study population in this outbreak is small. There are several confounding factors limiting this to a descriptive case series. Faecal microbiota has been shown to reflect the large intestinal microbiota but do not represent changes directly.

**Conclusions:**

These findings suggest that acute larval cyathostominosis is associated with dysbiosis of the gut microbiota as well as the inflammatory stimulus of numerous emerging larvae leading to structural and functional pathology of the large intestine.

## 1 INTRODUCTION

Cyathostomins are pervasive parasitic helminths, currently considered to be one of the most pathogenic endoparasites of equids.^1^, ^2^, ^3^, ^4^ There are over 40 species of cyathostomins that infect horses and they are ubiquitous in grazing horses, with prevalence of 89%‐100% reported in the literature.^4^, ^5^, ^6^, ^7^ Infection occurs through the faecal oral route, and larval stages of the parasites travel to the caecum and colon where they encyst in the intestinal mucosa and may become inhibited for up to three years before re‐emerging to mature to adult worms.^8^

Heavy burdens accumulating in the large intestinal lumen and mucosa result in the development of clinical disease which can manifest in several different forms^9^ including ill‐thrift and weight loss, with or without diarrhoea,^10^, ^11^ colic associated with caecal compromise^11^, ^12^, ^13^, ^14^ or a severe local and systemic inflammatory response syndrome termed acute larval cyathostominosis.^14^, ^15^, ^16^ Acute larval cyathostominosis is associated with the emergence “*en masse*” of previously encysted larvae from the mucosa of the colon and caecum resulting in severe typhlocolitis. Acute larval cyathostominosis has a high fatality rate with up to 50% mortality reported.^13^, ^14^, ^16^

In the literature, acute larval cyathostominosis is noted as having an obscure pathogenesis with reference to many risk factors age, season and period since last anthelmintic treated^17^ but also altered host immunity, stress, dietary changes and high intestinal helminth burden.^10^, ^16^, ^18^ It most commonly develops in late winter/early spring.^13^, ^16^, ^17^, ^19^ However, not all horses with recognised risk factors develop the disease and not all horses who develop acute larval cyathostominosis have these risk factors.^10^, ^20^ Therefore, it is recognised that other, as yet unknown, factors contribute to individual susceptibility to acute larval cyathostominosis.

The immunoregulatory effects of helminths in a wide variety of host species (human and animal). commonly involve a combination of Th2 and Treg responses (reviewed in^21^). No such studies have yet been carried out in horses. Furthermore, the treatment of helminth infections has been shown to influence the inflammatory dynamics of the host's immune response.^22^, ^23^, ^24^, ^25^ Although not many studies have evaluated the effects of cyathostomins on the immune response in horses, there is some evidence of a similar immunoregulatory response.^26^ In fact, it has already been hypothesised that removal of adults could restore a previously suppressed immune response^10^ and it has been shown that anthelmintic treatment in horses with a substantial helminth burden is associated with an inflammatory response.^27^, ^28^, ^29^ Moreover, there is growing evidence that helminths interact with the intestinal microbiota in horses.^29^, ^30^, ^31^ Dysbiosis of the microbiota has been shown to be associated with colitis^32^ and to precede colic.^33^ The role of intestinal microbiota in the development of acute larval cyathostominosis has not been well‐described.

Here, we describe an outbreak of acute larval cyathostominosis during November and December 2018 in a herd of horses on an equine rescue facility. The clinical presentation, clinicopathological parameters and gut microbiota analysis of the clinically affected horses, their clinically normal pasture mates and the post‐mortem findings on the non‐surviving cases are presented. There has been no case series to date that has outlined the changes in the faecal microbiota associated with this disease. This report suggests potential new approaches to understanding the complex tripartite relationship between the equine faecal microbiota, the helminth population (helminthome) and host immune responses in acute larval cyathostominosis.

## 2 MATERIALS AND METHODS

### 2.1 Study population

This outbreak occurred in the midlands of Ireland, in mid‐November 2018. The study population consisted of 23 mixed breed horses, mostly made up of cob ponies, large cobs and ponies, with ages ranging from 1.5 years to 12 years of age, 16 males, and 7 females. The horses had been rescued from various locations around the midlands by a charity organisation and it was planned to rehome them in time. Animals had been individually introduced into the herd over a 2‐year period prior to the outbreak (range: 2 weeks to 2 years). The group were kept on a 15‐acre plot of land that was considered an out‐farm. They were fed hay from the ground, as little grass was available, and had free access to water. The horses in this herd were selected for the pasture because they were considered “good doers” i.e. maintain and gain weight easily. Horses were introduced and removed from the herd regularly without quarantine protocols. No pasture faecal decontamination processes were employed, and no co‐grazing practices with other species were used. Anthelmintic treatment prior to the outbreak was intermittent and inconsistent; a variety of anthelmintic drugs had been used from 12 months to 3 weeks prior to onset of clinical signs.

Due to the high level of pasture contamination, the time of year and the age demographic of the horses, these horses were deemed an at risk population for acute larval cyathostominosis.^17^ Therefore, horses were suspected of having clinical cyathostominosis if they presented with the following clinical signs that were shown to be associated with acute larval cyathostominosis, acute weight loss, diarrhoea/soft faeces, pyrexia, dullness and colic.^13^, ^14^, ^16^, ^18^, ^19^, ^34^ The horses showing clinical signs were moved to individual stables on the main farm to prevent further exposure to infective larvae and to facilitate monitoring and treatment. The rest of the herd remained on pasture due to restricted land availability despite the risks associated with such highly contaminated pasture.

#### 2.1.1 Treatment and clinical monitoring

Clinically affected horses were treated on the main farm with anthelmintics, anti‐inflammatory drugs and supportive therapy as appropriate for each case. They were monitored continuously in their individual stables on the main farm. Monitoring included estimated feed and water intake, faecal consistency, demeanour and daily rectal temperature measurement. The animals had a full clinical examination performed by a veterinary practitioner on weekly visits or more frequently when deemed necessary due to deterioration of clinical status.

#### 2.1.2 Haematology and biochemistry

Blood samples were taken by jugular venepuncture into plain, EDTA, lithium heparin or sodium citrate Vacutainer® tubes as appropriate. Samples were collected from horses when clinical findings consistent with acute larval cyathostominosis first presented and thereafter were collected weekly from affected horses to monitor response to treatment. Blood samples were also taken from clinically normal horses under five years old that were co‐grazing on the out‐farm, as these were considered an at risk population.^17^ Haematological analysis was performed on blood samples collected in EDTA. Total protein, albumin and globulin concentration were measured in blood samples collected in lithium heparin. Fibrinogen concentration was measured in blood samples collected in sodium citrate and ELISA for encysted cyathostomins in samples collected in plain Vacutainer® tubes.

#### 2.1.3 Small redworm blood test

Blood samples were left to coagulate at room temperature for 24 hours prior to centrifugation at 1000g for 10 minutes. The serum was removed and stored at −20°C. A commercialised cyathostomin‐specific enzyme linked immunosorbent assay (ELISA) (Austin Davis Biologics Ltd) was performed as per manufacturer technical guidelines (www.austindavis.co.uk). This test detects IgG (T) antibodies specific to a combination of larval cyathostomin antigens from three common cyathostomin species.^35^ Results are reported as ‘Serum Scores’ which are relative concentrations of specific IgG (T) derived from ELISA absorbance and the use of ELISA calibration curves. Serum scores are reported together with statistically derived probabilities (using logistic regression models) that a horse is infected with a cyathostomin burden greater than a given threshold.

#### 2.1.4 Faecal sampling

Faecal samples were taken from horses who presented with clinical signs of acute larval cyathostominosis and from pasture mates, which were of similar age and clinically normal.

For faecal egg counts, fresh naturally voided faecal samples of at least three faecal balls were collected into zip lock bags if the dung was formed; loose faecal samples were collected into a faecal pot; all samples were stored at 4°C within 6 hours of collection, and subsequently examined for nematode eggs by the McMaster technique with an detection limit of 50 eggs per gram at a specific gravity of 1.2.^36^

For faecal microbiota analysis, fresh naturally voided faecal samples were collected into sterile containers. The middle of a faecal ball or uncontaminated top layer of diarrhoeic samples was collected and put on ice. The faecal samples were then stored at −20°C within 6 hours of collection.

#### 2.1.5 Faecal sample preparation and 16S rRNA gene sequence analysis

Faecal samples for microbiota analysis were processed as described previously.^29^ Briefly, faecal samples for microbiota analysis were homogenised and processed using mechanical and chemical lysis. DNA was extracted using the QIAamp® PowerFecal® Pro DNA Kit (QIAGEN®). DNA concentration was normalised and 16s metagenomic libraries were prepared using primers to amplify the V3‐V4 region of the bacterial 16s rRNA gene, with Illumina adaptors incorporated as described in the Illumina 16s Metagenomic Library Preparation guide. Following index PCR and purification, the products were quantified using the Qubit high sensitivity DNA kit (Life Technologies) and pooled equimolarly. The pooled libraries were assessed using an Agilent high sensitivity DNA kit and quantified by quantitative PCR (qPCR) using the Kapa Quantification kit for Illumina (Kapa Biosystems, USA) according to the manufacturer's guidelines. Libraries were then diluted and denatured following Illumina platform guidelines and sequenced (2 × 300 bp) on the Illumina MiSeq platform.

The sequences obtained were filtered on the basis of quality (removal of low‐quality nucleotides at the 3’ end and removal of window 10 nt with low average quality) and length (removal of sequences with less than 200 bp with prinsEquation ^37^, and the paired‐end reads with a minimum overlap of 50 bp were joined using Fastq‐join.^38^ Finally, all single files were processed to a final filtering sequence mean quality score >25 bp. The sequences were cleaned of replicates, and unique sequences and chimeras were checked against the GOLD database (https://gold.jgi.doe.gov) using the closed reference Usearch v7.0 algorithm.^39^ The resulting sequences were matched at operational taxonomic unit level (OTU; with 97% identity level) using UPARSE‐OUT algorithm with Usearch v7.0 algorithm. The taxonomic assignment of these OTUs was matched to results in the Ribosomal Database Project.^40^ Alpha and Beta diversities were determined using QIIME.^41^ Beta‐dispersion was quantified with betadisper (vegan::betadisper) and additional analyses were performed with the R package phylosEquation.^42^

#### 2.1.6 Post‐mortem examination

Post‐mortem examination was performed on horses who were euthanised. Intestinal tissue samples from the ventral and dorsal colon, caecum and small intestine, were fixed in 10% neutral buffered formalin. Following fixation, tissues were embedded in paraffin wax, sectioned at 4μm and stained with Gill®‐2 Haematoxylin and Eosin (H&E) for histopathological examination.

### 2.2 Data analysis

Statistical analyses were performed and graphical outputs of 16s rRNA gene high throughput sequencing data were generated with various packages in R.^42^ For beta‐diversity analysis, dissimilarity matrix between samples was calculated with the Bray‐Curtis method transforming the data into a logarithmic scale, studying the effects on microbiota composition. Variability between samples by Permutational Multivariate Analysis of Variance was calculated using Distance Matrices (Vegan *adonis* function including the variables of sex and antibiotics) from the R Vegan package.^43^ For alpha‐diversity values, assumption of normality was checked using the Shapiro‐Wilk test. Potential differences in alpha‐diversity included in the study were thereafter estimated by repeated measures analysis of variance (ANOVA), and t‐tests. Linear Discriminant Analysis Effect Size (LEfSe) was performed in order to discover specific bacterial biomarkers associated with health and disease states. Statistical differences between multiple samples at Phylum, Family and Genera levels were determined by Kruskal‐Wallis or Mann‐Whitney U‐tests, adjusting for multiple testing^44^ with the R statistical package (https://www.r‐project.org/). Statistical significance was established at *P* < .05.

Student T‐tests were used to determine differences in haematological parameters or plasma protein concentrations between the clinically normal horses and the clinically affected horses at first presentation Paired T‐tests were used to investigate changes in these parameters in repeated samples in the treated horses in the weeks after treatment. Statistical significance was established at *P* < .05.

## 3 RESULTS

### 3.1 Clinical presentation

**Figure 1:**
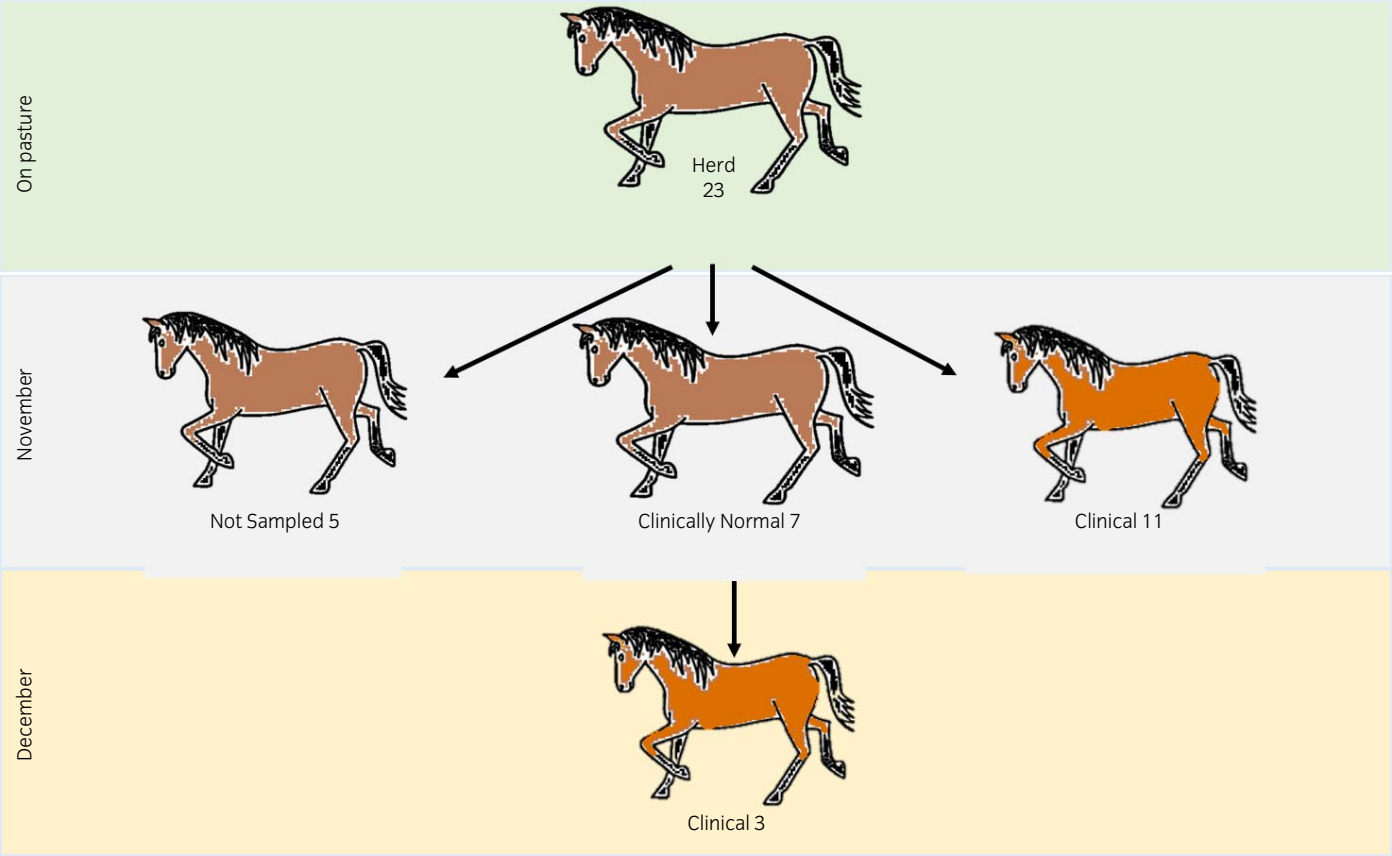
Schematic outline of the study population and timeline of outbreak. “Clinical” – horses who presented with clinical signs. “Not sampled” – horses who were not considered in the at‐risk cohort. “Clinically normal” ‐ horses that were considered at risk and had clinicopathological findings of chronic inflammation. “On pasture” – before outbreak, “November” and “December” timeline during the outbreak

Of the 23 horses grazing on the out‐farm where the outbreak occurred, 14 showed overt clinical signs consistent with acute larval cyathostominosis (9 males and 5 females; 1.5‐6 years old). Clinical signs appeared in 11 of the horses (8 males and 3 females; 1.5‐6 years old) over a period of one week. Three further horses presented with acute signs four weeks later (1 male and 2 females; 3‐4 years old) (Figure 1). There were a variety of clinical signs, with the most common being loose faecal consistency, weight loss, and pyrexia (Table S1). Loose faecal consistency was seen in 11 cases and ranged from transient, with resolution with 24 hours, to more chronic, persisting for five days post‐treatment. Weight loss was seen in nine cases, three of which presented with marked weight loss only. Persistent pyrexia (4‐10 days) was observed in five horses. Pyrexia resolved after anthelmintic treatment in three of the five horses. In the other two, pyrexia persisted after faecal consistency normalised, and did not resolve until antimicrobial therapy was instigated.

One horse, a 4‐year‐old gelding, presented with diarrhoea and pyrexia, which resolved after anti‐inflammatory and anthelmintic treatment. Three days after treatment he re‐presented with signs of moderate colic and pyrexia. He was referred to the University College Dublin Veterinary Hospital (UCDVH) for observation and received five days of fenbendazole (10 mg/kg bwt PO Panacur 10% (Intervet)). However, pyrexia persisted until antimicrobial therapy was instigated. Another 4‐year‐old gelding presented with loose dung, weight loss and pyrexia which resolved after anti‐inflammatory and anthelmintic treatment. Six and eight days, respectively, after treatment he developed pyrexia and preputial vasculitis. No cause for the recurrent pyrexia and vasculitis was determined and both resolved with broad spectrum antimicrobial therapy.

### 3.2 Anthelmintic treatment history

The prior anthelmintic treatment of the herd was inconsistent (Table S1). However, the seven most severely affected horses had been treated with an ivermectin or a combination of ivermectin/praziquantel product three weeks prior to first observation of clinical signs (Table S1). Three horses that had weight loss only had been dosed with ivermectin 12 weeks prior to presentation.

### 3.3 Treatment

The first four horses to present were initially treated by a veterinary practitioner who was not part of the study team, with a single dose (10 mg/kg) bwt of fenbendazole and a single dose (25 gs) of a trimethoprim/sulphonamide product (Table S1). This treatment was immediately discontinued to allow for assessment of the horses but occurred one day prior to faecal sampling in three horses; the fourth horse was euthanised and not included in the faecal microbiota sampling. These horses were then treated according to the authors’ protocol outlined below. The clinically affected horses were treated with oral prednisolone (1 mg/kg bwt PO SID Prednisolone 5 mg (Clonmel Healthcare, Gurtnafleur)) for 24‐48 hours prior to administration of moxidectin (0.4 mg/kg bwt PO Equest (Zoetis)). Three of those affected were subsequently treated with a five‐day course of fenbendazole (10 mg/kg bwt PO Panacur 10% (Intervet)) to cover for ascarid infection, two because of their age profile ie younger than 2 years of age, and one after referral to the UCDVH.

Supportive therapy in the form of oral fluids and electrolytes was administered twice daily in accordance with bodyweight. Non‐steroidal anti‐inflammatory drugs (flunixin meglamine (Finadyne (Bimeda)) 1.1 mg/kg bwt IV or phenylbutazone (Equipalazone (Interchem)) 4.4 mg/kg bwt PO) were administered if the horse had persistent pyrexia ie temperature above 38.5°C for 3 days or more or if the temperature was above 40°C, with anorexia.

Broad spectrum oral antibiotics (enrofloxacin (Kariflox (Bimeda)) 7.5 mg/kg bwt PO SID and metronidazole (Flagyl (Uniphar)) 25 mg/kg bwt PO BID), were administered to four horses. These horses had persistent pyrexia despite steroid and NSAID administration and clinical and clinicopathological evidence of endotoxaemia and/or other complications (vasculitis/colic). After initiation of antimicrobial therapy, the horses’ demeanour improved, and they became normo‐thermic within 1‐3 days.

### 3.4 Haematology and biochemistry

Blood samples were collected within one day of first presentation of clinical signs in all but two of the 14 clinically affected horses (12). Repeated sampling was performed after treatment in accordance with clinical status and thus not all horses were sampled on each occasion. Eight horses had blood samples taken 15‐17 days post treatment, and five of these had blood samples again at 22‐24 days post‐treatment. Blood samples were also taken from seven horses younger than four years who were clinically normal at the time of sampling (Table S1). Three of these horses presented with weight loss 26 days later, blood samples were taken at first clinical presentation and were included in the clinically affected group.

Consistent findings in blood samples from all clinically affected and clinically normal horses included borderline anaemia, leucocytosis, thrombocytosis, lymphocytosis, hyperfibrinogenaemia, hyperglobulinaemia and a reverse albumin: globulin (A: G) ratio (Table S2). Clinically affected horses had higher neutrophil counts (*P* = .01) and lower albumin (*P* = .002) and total serum protein (*P* = .02) concentrations than clinically normal horses. Total serum protein concentrations, however, remained within the reference range. The more severely affected cases presented with neutrophilia with a left shift and monocytosis.

Following treatment, the left shift took up to two weeks to resolve. There was no significant difference in total white blood cell or neutrophil counts in repeated samples. Monocyte numbers were not significantly different at 15‐17 days but were significantly lower at 22‐24 days. However, they remained increased at 22‐24 days in individual horses. Lymphocyte numbers decreased post treatment at 15‐17 days (*P* < .01) and 22‐24 days (*P* < .03). Significant decreases were seen at 15‐17 days in packed cell volume (*P* < .01) and platelet counts (*P* < .03), but by 22‐24 days, there was no significant difference from baseline.

Total proteins concentrations were decreased at 15‐17 days (*P* < .02) and 22‐24 days (*P* < .03) post treatment. Hypoalbuminaemia persisted post‐treatment and was not significantly different from baseline at 22‐24 days. Globulin concentrations decreased significantly 15‐17 days post treatment (*P* < .04) but were not significantly different from pretreatment concentrations at 22‐24 days; the A:G ratio remained low. Fibrinogen concentration remained above normal at 22‐26 days post‐treatment and was not significantly different in repeated samples.

### 3.5 Small redworm blood test

Six of the clinically affected horses were included in this analysis. All horses had very high Serum Scores, ranging from 53.7 to 70.9 therefore indicating a 76.9% to 91.7% (respectively) probability that the horses had > 10 000 total worm burden^35^ (www.austindavis.co.uk).

### 3.6 Faecal egg counts

Faecal samples were obtained at presentation from all clinically affected horses (except horse 11) and four clinically normal pasture mates. Only one clinically affected horse had a faecal egg count of over 200epg at presentation (Table S1). In the horses treated with moxidectin, FEC were also taken two weeks after treatment to ensure efficacy of treatment and were negative.

### 3.7 Faecal microbiota analysis

Prior to the implementation of the authors’ treatment protocol, faecal microbiota samples were analysed in 10 of the 14 clinically affected horses at presentation (two horses had been euthanised and two horses were only transiently affected). At that time, by way of creating a comparison group, faecal samples were taken from seven clinically normal pasture mates. Three of these pasture mates developed clinical signs (acute weight loss) four weeks later. Accordingly, samples were taken at the time of the development of clinical signs and included in the clinically affected group for analysis (Figure 1).

**Figure 2:**
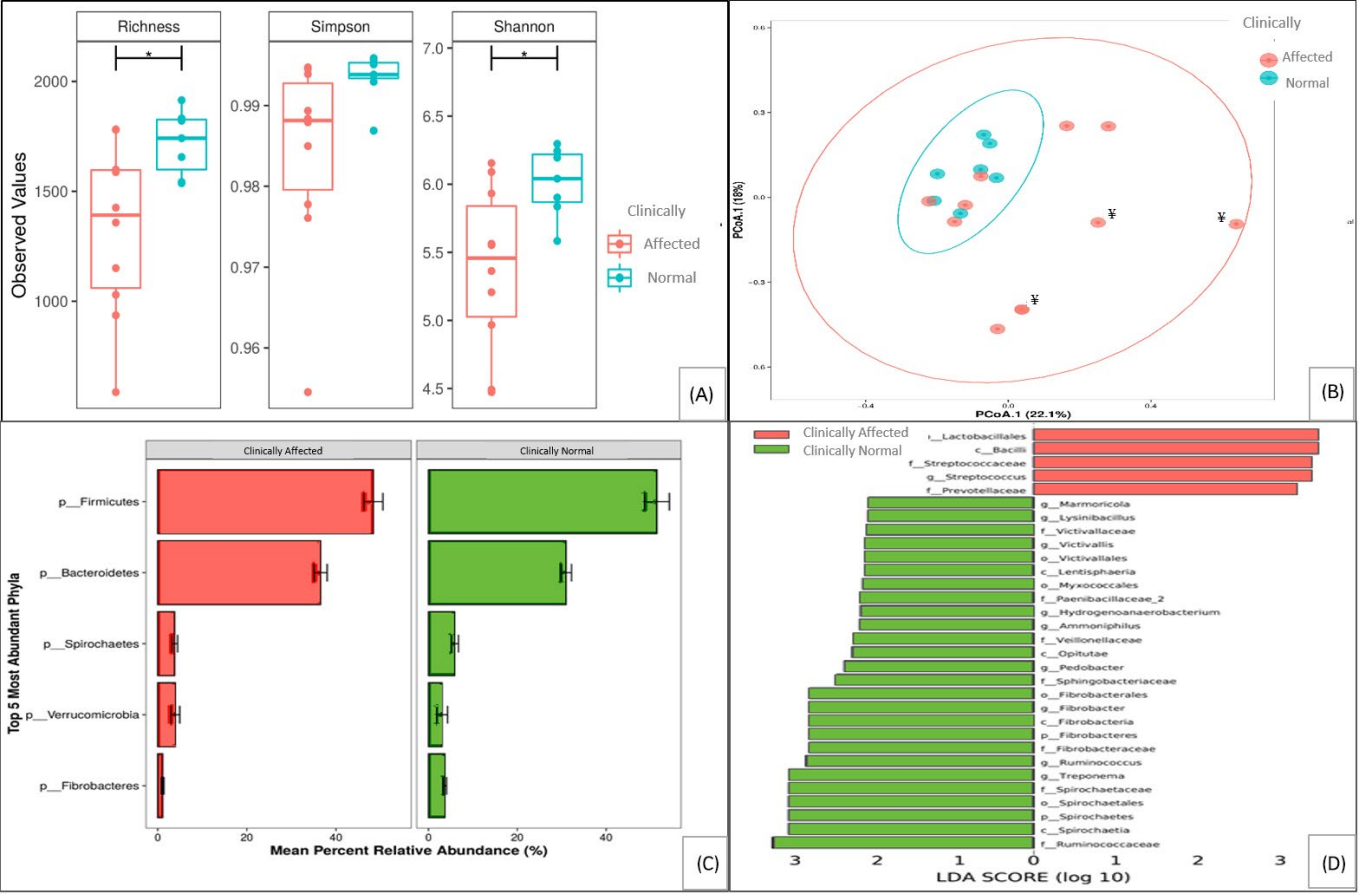
Faecal microbiota characteristics in horses from the study group where sampling was carried out at first presentation of clinical signs (n = 10), or which were only clinically normal/developed clinical signs later in the outbreak after faecal microbiota analysis (n = 7). (A) Alpha‐diversity measurements of faecal microbiota by Richness, Simpson and Shannon indices (B) Beta‐diversity measurements of faecal microbiota by PCoA. ¥ denotes horses who received antibiotic and anthelmintic treatment one day prior to sampling (C) Relative abundance of faecal microbiota. Phyla are displayed as mean percent relative abundance. (D) Comparison of the two groups by linear discriminant analysis (LDA) effect size (LEfSe) algorithm. Note: sampling was not done on the two horses who were euthanised and two horses that were mild and transiently affected. Significant differences are indicated: *p < 0.05. For (B) P = 0.015

A comparison of the faecal microbiota, using 16s rRNA sequencing between horses who presented with clinical signs and their grazing cohorts who were not clinically affected, revealed differences in species richness and diversity. The clinically affected horses had a decreased alpha‐diversity when compared to the clinically normal group when assessed using the OTU Richness (*P*.value = .03; F.value = 6.064) and Shannon index (*P*.value = .02; F.value = 6.755) (Figure 2a).

In order to ensure that beta diversity estimates were not affected by the administration of antibiotics, we excluded the three animals that had received treatment prior to sampling from this analysis. We also included sex as a possible variance factor before investigation the clinical factor of interest.

Beta‐diversity measurements showed a significantly increased dispersion (*P* = .02) of the clinically affected horses when compared with clinically normal pasture mates (Figure 2b). The major Phyla detected were similar in the two groups (Figure 2c). The Phyla Firmicutes and Bacteroidetes were the most abundant, followed by Spirochaetes, Verrucomicrobia and Fibrobacteres.

To identify statistical and specific differences in the communities of microbiota associated with disease, we used the linear discriminant analysis (LDA) effect size algorithm (LEfSe) that reflects increases in specific members of a Phylum, Class, Order, Family and Genus (Figure 2d). In the clinical group there was a greater relative abundance of the class Bacilli, the order Lactobacillales, the family Streptococcaceae and genus *Streptococcus* and the Prevotelleceae family. In the clinically normal group, there was a greater relative abundance of several groups, most notably in the obligate fibrolytic bacteria i.e. the phylum Fibrobacteres, the order Fibrobacterales, class Fibrobacteria, genus *Fibrobacter* and the family Ruminococcaceae, genus *Ruminococcaceae* (Figure 2).

### 3.8 Outcome

In 12 of 14 clinically affected horses, clinical signs resolved with treatment and supportive care. Two horses (Case 1 and Case 2) required euthanasia due to the severity of their clinical signs. Case 1 presented with acute diarrhoea and weight loss and was euthanised at the UCDVH shortly after admission on Day 2 of the outbreak. The severity of the presentation was exacerbated by head trauma causing severe cellulitis and affecting prehension and drinking ability. Case 2 presented with diarrhoea and pyrexia, followed by severe weight loss four days later and was euthanised at the UCDVH after 48 hours of intensive supportive care. Three horses lost considerable weight acutely again within 1‐3 months and needed subsequent treatment.

### 3.9 Post‐mortem findings

**Figure 3:**
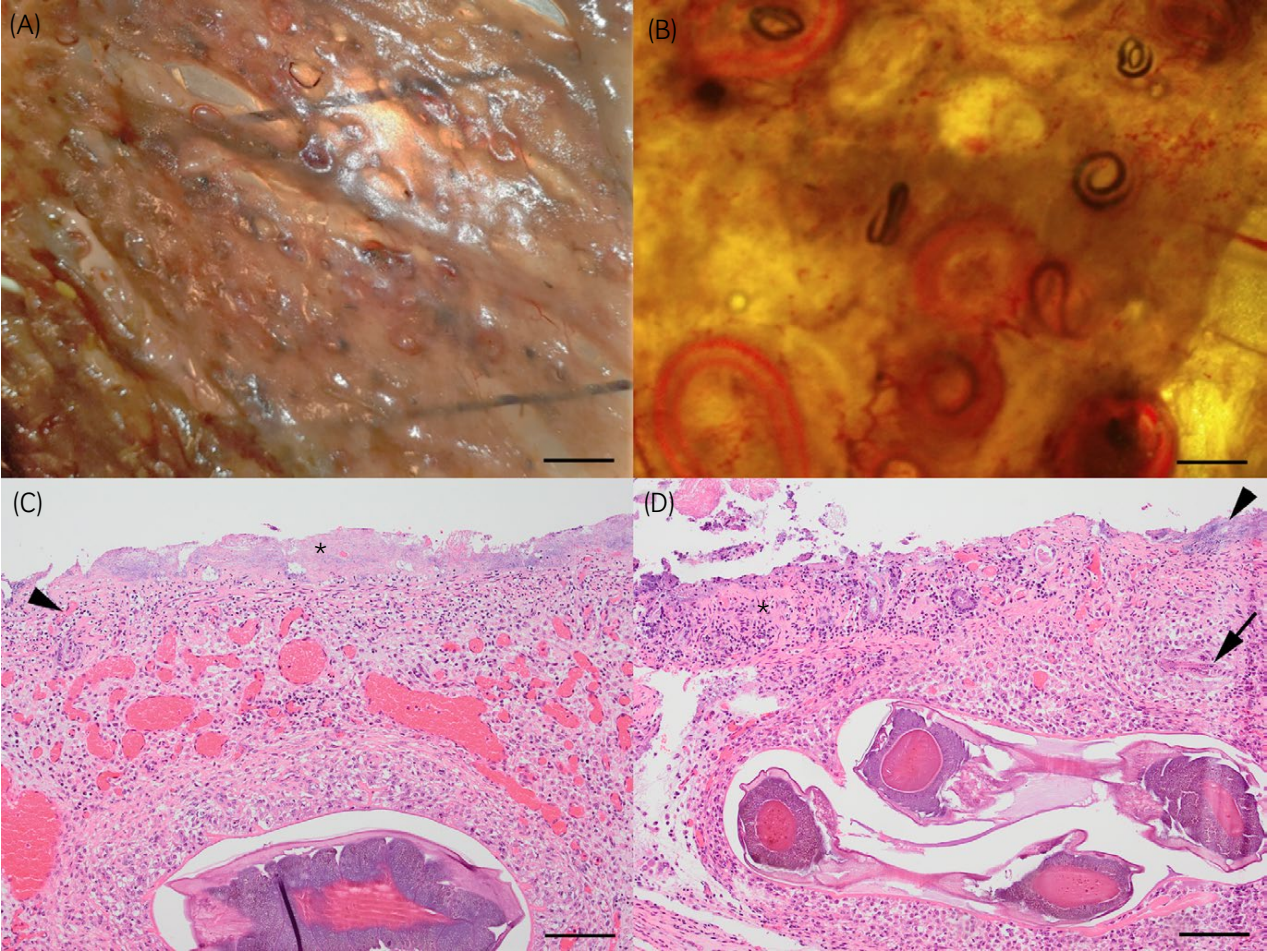
Intestine from 2‐year‐old horse with acute cyathostominosis (Case 2); Illumination of the mucosal layer of the ventral colon with large numbers of L4 cyathostomins in (A) bar = 2 cm, (B) bar = 1 cm. Photomicrograph of caecum (C) shows diffuse severe mucosal necrosis and loss with infiltration of large numbers of coccoid bacteria (asterisk). There is vascular thrombosis with infiltration of neutrophils (arrowhead). The submucosa is infiltrated by macrophages surrounding the encysted L4 and markedly dilated blood vessels are present. Similar changes are seen in the ventral colon (D) with marked mucosal necrosis (asterisk) and bacterial overgrowth (arrowhead). The submucosa is densely infiltrated by macrophages surrounding L3 (arrow) and encysted L4. Haematoxylin and Eosin stain, bar = 100 µm

Case 1 had remained in good body condition with visceral fat deposits. It had developed severe necrosuppurative cellulitis extending from the right nares over the maxilla to the right eye. Case 2 had lost considerable weight and was in poor body condition. In both horses marked changes were observed in the caecum and colon consisting of intestinal wall thickening with scattered multifocal, 0.2‐0.5cm diameter, round, white raised nodules and marked ulceration and necrosis associated with widespread haemorrhage. Trans‐illumination of the mucosal layer of the ventral colon revealed more than 400 encysted mucosal cyathostomin larvae per 8 × 8 cm^2^ sample (Figure 3, a, b).

Histopathological examination confirmed that the gross findings showing widespread multifocal to coalescing marked necrosis and ulceration of the entire mucosa (Figure 3 c, d). This was associated with infiltration of viable and degenerate neutrophils, large numbers of superficial bacterial colonies, multifocal vascular thrombosis and marked haemorrhage. Multiple L4 cyathostomin larvae were present in the submucosa either within a fibrous capsule or surrounded by macrophages (Figure 3c). Moderate numbers of much smaller less‐developed L3 larvae were seen surrounded by similar changes (Figure 3d). A diagnosis of severe necrotising typhlocolitis associated with cyathostomin larvae and bacterial overgrowth was made in both cases. An additional finding in the second horse was multifocal necrosuppurative hepatitis with intra‐lesional bacteria consistent with bacteraemia.

## 4 DISCUSSION

We report here on an outbreak of acute larval cyathostominosis in Ireland, with 14 of 23 horses showing overt clinical signs, of which two were euthanised and the other 12 recovered. This is the first time that clinical and clinicopathological parameters from presentation to clinical remission and faecal microbiota changes associated with the onset of disease have been documented in an acute larval cyathostominosis outbreak.

In this outbreak, as in other cases in the literature, there was a lack of pasture hygiene practices such as faecal removal, rest periods or cross grazing.^15^, ^19^ This pasture had more than the recommended one horse per 1‐2 acres^45^ with up to 25 horses on the 15 acre pasture at times. Furthermore, although these horses were in good condition, they were originally rescued on welfare grounds, often due to lack of concern for the animal's basic requirements including preventative healthcare. Therefore, it is likely that these horses would be at risk of accumulating high cyathostomin burdens prior to arrival on farm.

Additionally, no consistent anthelmintic regime had been in place. Although acute larval cyathostominosis can occur in regularly dewormed horses,^15^, ^19^, ^20^ long periods of time since last anthelmintic treatment is associated with increased risk of developing the disease^17^ most likely due to the build‐up of large intestinal larval burdens. Moreover, treatment efficacy and resistance must be considered. Fenbendazole and moxidectin have a reported efficacy against early stage 3 larvae (EL3) of 50.4% and 73.8%, respectively, and against late stage 3 larvae (LL3) and stage 4 larvae (L4) efficacy of 70.8% and 74.6%, respectively.^46^ There is growing concern, due to the decrease in egg reappearance time after seemingly effective moxidectin treatment (>95% FECRT), that resistance is building in some species.^46^, ^47^, ^48^, ^49^ In the past growing resistance to anthelmintics in cyathostomins was associated perceived increase in the incidence of ALC.^14^, ^50^

FEC were not routinely done on this herd, however, faecal samples would not have been an indicator of disease in this outbreak. On clinical presentation only one horse had a FEC above 200epg, a recommended treatment cut‐off,^51^ which highlights that FEC are not useful for identifying at risk horses or the disease process itself. This has consistently been reported in the literature.^11^, ^16^, ^19^, ^52^

The 14 clinically affected horses in the study had clinical signs and clinicopathological findings which varied in severity from acute signs of weight loss, diarrhoea and pyrexia, which are the most common signs of acute larval cyathostominosis described in referral hospital reports,^13^, ^14^, ^16^ to noticeable weight loss similar to that reported previously in other outbreaks.^15^, ^19^

Persistent pyrexia that was unresponsive to anthelmintic and anti‐inflammatory treatment was observed in horses with more severe clinical signs. Reilly^53^ described a resurgence of a pyrexia associated with endotoxaemia in a fatal case of acute larval cyathostominosis. The more severely affected horses with persistent pyrexia in this study also had clinical and clinic‐pathological evidence of endotoxaemia, such as injected mucous membranes and a left shift. Endotoxaemia most likely occurred from intestinal wall compromise; this was also suggested as a possible cause of endotoxaemia in a report of 12 cases of acute larval cyathostominosis in a referral hospital.^13^

All horses in the study that had haematology and biochemical analysis performed, including clinically normal horses, had clinicopathological evidence of chronic active systemic inflammation (hyper‐fibrinogenemia and reversed A:G ratio). The horses that presented with more severe clinical signs had clinicopathological changes suggesting an overwhelming inflammatory and protein losing condition. Eosinophilia was not always observed in the severe cases but was seen in clinically normal cases. These findings are in line with the clinicopathological findings in several other reports.^14^, ^15^, ^16^, ^19^, ^54^ Some blood parameters during the treatment and convalescent periods in this study showed transient improvement (monocytes) but many others (WBCC, platelet numbers, fibrinogen, globulins and albumin) remained abnormal even three weeks post‐treatment. Although the persistent neutrophilia and decrease in lymphocyte numbers over time could be attributed to the treatment with corticosteroids, the sustained increase in other inflammatory markers, such as fibrinogen and the A:G ratio suggest that significant underlying inflammatory disease was still present. This has been reported previously, with an improvement in horses after initiation of treatment followed by a worsening or reoccurrence of signs and death in some cases.^10^, ^15^, ^53^ We suggest that in this herd, horses were all affected with chronic cyathostominosis, but that individual horses suffered from different intensities of disease, with some remaining clinically normal. Those horses that presented with severe clinical signs were either overwhelmed by infectious burdens or had experienced a trigger to induce synchronised larval re‐emergence and an acute severe typhlocolitis.

The cause of the severe post‐mortem changes in the two horses which were euthanised is unclear. The mucosal changes are in general similar to those described in other reports.^16^, ^19^, ^53^ However, the bacterial over‐growth has not been previously described and is an indication that a disruption in the bacterial population could be a contributing factor to the deterioration of some cases. The severe disruption of the mucosal barrier in combination with the profound inflammatory response and bacterial overgrowth provides a reason for the endotoxic signs associated with the most severely affected horses.

Corticosteroids in conjunction with anthelmintic treatment, most often moxidectin, are recommended for the treatment of larval cyathostominosis, together with supportive therapy.^18^, ^45^, ^52^, ^55^, ^56^ In addition to anthelmintic treatment with moxidectin, corticosteroids were administered to clinically affected horses and tapered slowly according to clinical response, and we did not observe any adverse effects attributable directly to the steroid use. Notably two horses, which showed only mild transient diarrhoea, did not receive corticosteroids; one of these horses re‐presented within four weeks with signs of acute severe weight loss. A combination of moxidectin and corticosteroids appears to be safe and effective in the treatment of horses suspected to be suffering from cyathostominosis and at risk of acute larval cyathostominosis.

In this study, we considered it appropriate to administer broad spectrum antibiotics to individual high‐risk cases i.e. persistent pyrexia and/or significant evidence of endotoxaemia (toxic mucus membranes, degenerative/toxic left shift). This protocol was influenced by post‐mortem examination findings of the two euthanised horses, where severe colonic wall destruction and compromise of the intestinal barrier made bacterial translocation very likely. Antibiotics have been used in cases of acute larval cyathostominosis as documented in the literature^13^, ^16^, ^19^ and may be indicated in cases with clinical evidence of endotoxaemia.

Our findings in this case series are in line with the growing evidence from both animal and human studies, that there is a relationship between parasitic helminths, the intestinal microbiota and the host immune response.^57^, ^58^, ^59^, ^60^, ^61^, ^62^ There was a significant decrease in alpha‐diversity of faecal microbiota in the clinically affected horses when compared to their clinically normal pasture mates. In our recent study, regarding the effects of anthelmintic use on clinically normal horses, we reported a decrease in microbiota diversity and a concomitant inflammatory response^29^ which is line with findings in humans that showed the effects of anthelmintic treatment.^63^ A decrease in alpha‐diversity has also been shown in colitis^32^ and colic^33^ in the horse. In addition, beta‐diversity analysis showed an obvious increased dispersion of the clinically affected horses, reflecting the dramatic changes to their microbiota when compared to their clinically normal co‐grazing herd‐mates. Although three of these horses received antibiotic and anthelmintic treatment the day before sampling, these changes were also evident in the other clinically affected horses.

In horses with cyathostomin burdens, anthelmintic treatment is primarily effective against adult worms in the gut lumen, and removal of the adults is likely to break a negative‐feedback loop that is suspected to prevent hypobiotic larvae resuming development and emerging into the lumen.^8^, ^64^ While some emergent larvae will be killed by anthelmintic treatment, we hypothesise that the dominant effect in this situation is a concatenation of inflammation arising from removal of adult cyathostomins and a wave of emerging larvae, whilst also disturbing the delicate balance between the helminth and host, and in particular the potentially beneficial anti‐inflammatory influence that prevails in steady state. The inflammatory profile of the horses in this acute larval cyathostominosis outbreak, with features associated with gut microbiota dysbiosis as well as intestinal and systemic inflammatory responses, is consistent with this hypothesis.

The differences between communities of bacteria associated with clinically affected and clinically normal horses assessed using the LefSe algorithm, were interesting but not conclusive. *Prevotella* is considered a commensal and is associated with the colonic microbiota community.^65^ Although, some *Prevotella* strains have been shown to exhibit pathobiontic properties in humans and mice [reviewed by 66] only a very tenuous association with colitis was seen in horses.^32^The increase in the genus *Streptococcus* most likely lead to the increase in the corresponding family, order and class. Although *Streptococcus* is considered a commensal organism in horses,^67^, ^68^ it is also considered an opportunistic bacteria that has been associated with the equine carbohydrate overload colitis model that induces acute laminitis,^69^ chronic laminitis^70^ and with horses with colic.^71^ There was a decrease in abundance of *Fibrobacter* in clinically affected horses. These are cellulytic bacteria associated with the core microbiome of horses^72^ and decrease with dietary change and intestinal disease.^73^ *Ruminococcus,* another predominant cellulytic bacterial group, was decreased in abundance with similar changes been associated with colonic disease in horses.^32^, ^33^ This combination of an increase in *Streptococcus* with a corresponding decrease of obligate fibrolytic bacteria has been linked previously with equine intestinal disease.^73^ The most common presenting sign in the horses in this outbreak was weight loss, and in fact, weight re‐gain was the longest aspect of recovery, despite the majority of cases maintaining a normal appetite throughout the disease process, which has been seen in other acute larval cyathostominosis cases.^19^ This could be due to structural damage to the intestinal wall and possibly dysbiosis causing a reduction in essential bacteria for hind gut fermentation and digestion. All horses were on the same pasture at the time of the outbreak and were fed supplementary hay due to the poor quality of pastures. Clinically affected horses were stabled after diagnosis, and hence the changed environment cannot be ruled out as contributing to microbiota changes. However, rapid weight loss is a cardinal sign of acute larval cyathostominosis^18^ and therefore it is likely that the microbiota changes observed are directly or indirectly related to these changes. This association is in need of further study.

The study population in this outbreak is small; however, it does represent a relatively large cohort that was simultaneously affected. Although other causes of a chronic protein losing enteropathy (PLE) were not actively ruled out, in the face of the occurrence of this disease in multiple horses, other PLE aetiologies, such as IBD and alimentary lymphoma,^74^ which tend to occur in individual cases, were considered highly unlikely. Bacterial aetiologies of colitis, such as *Salmonella* and *Clostridia*, most commonly are per‐acute in onset with rapid progression and bacterial enteritis, such as *Lawsonia intrcellularis*, was excluded due the demographic of horses affected, i.e. 2‐6 years old, with it most commonly affecting weanlings.^75^ Viral causes of colitis such as Corona Virus, were considered unlikely; only a small number individual cases have been diagnosed in Ireland and it more commonly presents with lethargy, anorexia and fever, less frequently with GIT related signs.^76^ The faecal microbiota has been shown to reflect the large intestinal microbiota, but of course does not represent changes directly.^67^ However, faecal sampling is readily accessible and non‐invasive, and thus a valuable tool in investigations of disorders of the equine gut, and particularly the large intestine. Three of the clinically affected horses had been treated with one dose of antibiotics and anthelmintics one day prior to initial faecal sampling. We acknowledge that this treatment may have perturbed the faecal microbiota in these animals.

## 5 CONCLUSION

Together with our previous study^29^ and findings described here of the severe typhlocolitis, marked weight loss and endotoxaemia seen in some of the cases in this outbreak, we suggest that the systemic inflammation and acute clinical syndrome that is seen in acute larval cyathostominosis could arise from the “perfect storm” of an inflammatory stimulus of numerous emerging larvae and dysbiosis of the gut microbiota leading to structural and functional pathology of the large intestine. Further investigation into the relationship between cyathostomins, the gut bacterial microbiota and inflammatory changes in normal, sub‐clinical and clinically affected horses will be useful in developing biomarkers that can be used for risk assessment and diagnosis.

## ETHICAL ANIMAL RESEARCH

Research ethics committee oversight not required by this journal: retrospective analysis of clinical data.

## OWNER INFORMED CONSENT

The data used in this study was collected form clinical samples taken during the course of the disease outbreak.

## CONFLICT OF INTERESTS

No competing interests have been declared.

## AUTHORSHIP

N. Walshe participated in the study design, execution, data analysis and interpretation and preparation of the manuscript. V. Duggan participated in the study design, execution, data analysis and interpretation, preparation of the manuscript and approved the final version of the manuscript. G. Mulcahy participated in study design, execution, data analysis and interpretation, preparation of the manuscript and approved the final version of the manuscript. H. Jahns performed the post‐mortem analysis and contributed to preparation of the manuscript. P. Cotter, F. Crispie and R. Cabrera‐Rubio carried out the 16S rRNA gene sequence analysis and related analysis, and contributed to preparation of the manuscript.

### Peer Review

The peer review history for this article is available at https://publons.com/publon/10.1111/evj.13350.

## Supporting information

Table S1

Table S2

## Data Availability

The data that support the findings of this study are available on request from the corresponding author. The data are not publicly available due to privacy or ethical restrictions.
