# Supplementary material for: Outbreak of acute larval cyathostominosis – A “perfect storm” of inflammation and dysbiosis
Source: Equine Vet J. 2020 Oct 6;53(4):727–39. doi: 10.1111/evj.13350 (PMC8246859; doi:10.1111/evj.13350)
Supplement: Supplementary file 2 — Table S2 [file EVJ-53-727-s002.pdf]

Equine Veterinary Journal BEVA

Table S2: Haematology and serum biochemistry in sub-clinically affected (a) and clinically affected (b) horses. Clinically normal n=7 (3 of which developed clinical signs later in the outbreak) and Clinical n= 12 (samples taken at presentation of clinical signs and at 16 and 23 days post-initiation of treatment. Note: sampling was done in accordance with clinical status of horse on each visit and thus not all horses were sampled on each occasion. Figures in bold are outside the reference range. Hct = haematocrit; Plt = platelets; WBC = white blood cells; NeutA = neutrophils; Neut BA = band neutrophils; LymA = lymphocytes; MonoA = monocytes; BasaA = basophils; EosA = eosinophils; Fib = fibrinogen; TP = Total protein; Glob = globulin; Alb = Albumin; A:G = albumin:globulin.

			**Clinically normal**	**Clinical**		
				At presentation (n=12)	16d post tx (n=8)	23d post tx (n=5)
		**Reference range**	Mean (range) (n=7)	Mean (range)	Mean (range)	Mean (range)
**Hct**	l/L	0.29-0.44	0.35 (0.29-0.39)	0.35 (0.27-0.42)	0.32 (0.25-0.36)	0.32 (0.23-0.43)
**Plt**	10^9/L	116-189	**223 (200-318)**	**229 (64-397)**	**339 (229-425)**	**364 (311-446)**
**WBC**	10^9/L	4.37-8.96	**10.7 (7.4-15.5)**	**14.3 (8.5-27.9)**	**16.7 (9.6-22.9)**	**16.0 (10.3-19.6)**
**NeutA**	10^9/L	2.22-5.8	4.8 (2.8-6)	**8.6 (2.4-18.1)**	**9.2 (4.2-16.7)**	**10.3 (6.0-13.7)**
**NeutBA**	10^9/L		0.0	**1.0 (0.2-2.4)**	0 (0-0)	**1.0 (0.0-4.8)**
**LymA**	10^9/L	0.99-3.48	**5.0 (3.0-9.0)**	**4.4 (1.8-6.7)**	**6.8 (4.0-9.2)**	**5.8 (4.3-6.8)**
**MonoA**	10^9/L	0.04-0.4	0.3 (0.1-0.8)	**0.5 (0.1-1.4)**	0.4 (0.0-0.7)	0.1 (0.0-0.4)
**BasaA**	10^9/L	0-0.2	0.1 (0-0.3)	0.1 (0.0-0.3)	0 (0-0)	0.1 (0.0-0.4)
**EosA**	10^9/L	0-0.35	**0.5 (0.2-0.9)**	**0.4 (0.2-0.8)**	0.2 (0.0-0.6)	0 (0-0)
**Fib**	g/L	0-2	**2.3 (1.8-2.7)**	**2.8 (1.0-3.9)**	**2.6 (1.7-3.4)**	**2.3 (2.0-2.9)**
**TP**	g/L	57-79	**87.2 (71.7-99.6)**	75.4 (61.0-90.5)	66.9 (51.6-84.9)	63.1 (54.4-75.1)
**Glob**	g/L	24-48	**55.4 (39.1-67.9)**	**48.8 (35.1-64.0)**	41.9 (29.5-55.4)	38.3 (32.2-49.3)
**Alb**	g/L	29-37	31.8 (26.9-35.8)	**26.6 (24.2-31.9)**	**25 (20.9-29.5)**	**24.8 (20.6-28.1)**
**A:G**		0.62-1.46	**0.6 (0.5-0.8)**	**0.6 (0.5-0.7)**	**0.6 (0.4-0.8)**	0.7 (0.5-0.8)
