# Supplementary material for: Outbreak of acute larval cyathostominosis – A “perfect storm” of inflammation and dysbiosis
Source: Equine Vet J. 2020 Oct 6;53(4):727–39. doi: 10.1111/evj.13350 (PMC8246859; doi:10.1111/evj.13350)
Supplement: Supplementary file 1 — Table S1 [file EVJ-53-727-s001.pdf]

Equine Veterinary Journal BEVA

Table S1: Anthelmintic treatment history, clinical signs and outcome for 18 horses from the 23 horse study population - 14 clinically-affected and 4 “at risk” horses “†” showing only clinicopathologic abnormalities. The horses are ranked according to time since last anthelmintic treatment - showing time on pasture, time since last anthelmintic treatment, time since last larvicidal anthelmintic treatment, treated one day before sampling, faecal egg count (FEC) at presentation, clinical presentation and outcome; relapse was 1-3 months after presentation; received treatment pre-sample refers to anthelmintic and antibiotic treatment given before implementation of authors’ protocol.
SIRS = systemic inflammatory response syndrome; N/R = Not recorded; N/A = Not applicable.

**No**	**Age**	**Time on Pasture**	**Last Larvicidal Treatment**	**Last Anthelmintic Treatment**	**Received Treatment Pre-sample**	**FEC at presentation**	**Clinical Signs**								**Outcome**
	Yrs	Months	Months	Weeks		EPG	Diarrhoea	Loose dung	Weight loss	Pyrexia	Anorexia	SIRS	Colic	Vasculitis	
**1**	3	5	9	3		0	+		+			+			Euthanasia
**2**	2	1.5	9	3		50	+		+	+		+			Euthanasia
**3**	2	14	9	3	+	0	+		+	+	+	+			Remission
**4**	2	14	9	3		0		+	+	+	+	+			Remission
**5**	4	4	8	3		0		+	+	+			+		Remission
**6**	4	17	19	3	+	0		+	+	+		+		+	Relapse
**7**	4	3	12	3	+	0		+	+						Relapse
**11**	5	3	12	8		N/R		+							Remission
**14**	3	?	9	8		250			+						Remission
**12**	3	14	10	8		0			+						Remission
**13**	4	13	10	8		100			+						Remission
**10**	4	5	9	22		0		+							Relapse
**15†**	3	0.25	8	32		1000									N/A
**16†**	3	0.25	N/R	36		0									N/A
**17†**	6	2.5	13	36		200									N/A
**8**	6	10	9	39		10		+							Remission
**9**	5	14	9	39		100		+							Remission
**18†**	4	3	19	60		200									N/A
